# Influence of School-Based Smoking Prevention Education on Reducing Gap in Exposure to Anti-Tobacco Media Message among Korean Adolescents

**DOI:** 10.3390/ijerph17238742

**Published:** 2020-11-25

**Authors:** Jun Hyun Hwang, Dong-Hee Ryu, Soon-Woo Park

**Affiliations:** Department of Preventive Medicine, Catholic University of Daegu School of Medicine, Daegu 42472, Korea; pmdr213@cu.ac.kr (J.H.H.); ryudh@cu.ac.kr (D.-H.R.)

**Keywords:** adolescent, anti-tobacco media message, school-based smoking prevention education

## Abstract

Korean adolescents at high risk for smoking are less exposed to anti-tobacco media messages. This study examines whether school-based smoking prevention education is related to media exposure and whether it can contribute to reducing the gap in exposure to anti-tobacco media messages between smoking vulnerable and non-vulnerable groups. A nationally representative dataset, the 2018 Korea youth risk behavior web-based survey, comprising 59,410 students from grades 7–12, was analyzed. Logistic regression models were designed to evaluate the association between school-based smoking prevention education and media messages exposure. Within-group differences in exposure levels based on sociodemographic characteristics were compared depending on participation or nonparticipation in school-based smoking prevention education. Experience of smoking prevention education within a year was significantly associated with exposure to anti-tobacco media messages. Among Korean adolescents who participated in smoking prevention education compared to those who did not, the media messages exposure rate was more than 20% higher, and the exposure gap within the subgroups by sociodemographic characteristics was narrower. Participation in school-based smoking prevention education was significantly related to media messages exposure. This relationship can be used to improve the overall media messages exposure rate and to reduce the differences in exposure rate based on sociodemographic traits.

## 1. Introduction

Mass media campaigns placing health-associated messages on media, such as TV, Internet, radio, newspapers or magazines, are frequently used to prevent unhealthy behaviors and to promote positive changes in health behaviors [1]. Media campaigns on tobacco use influence smoking-related knowledge and norms at the individual and community levels. Persuasive messages contained in these campaigns can further help in preventing smoking in adolescents and inducing smoking cessation in adults [2,3,4]. Anti-tobacco campaigning is one of the major tobacco control policies of the World Health Organization (WHO) Framework Convention on Tobacco Control (FCTC) as listed in the guidelines for implementation of Article 12 on education, communication, training and public awareness [5]. It has also proven an effective measure for reducing tobacco demand introduced in one of the six policies of the MPOWER package, “Warn about the dangers of tobacco” [6].

An important process in planning health communications is determining a spectrum of target audiences, whether the general public or a specific group [7]. Although mass media campaigns can deliver anti-tobacco messages to a general audience, segmentation of the target group and focusing on high-risk groups—those who are likely to initiate smoking—is a more practical strategy. If media campaign strategies targeting high-risk groups are not effective, the smoking prevalence gap between the high-risk group and the low-risk group may widen further by failing to prevent or delay the initiation of smoking in the high-risk group. In the Real Coast campaign aimed at the youth in the United States, the awareness of anti-tobacco media was higher among cigarette experimenters, higher graders, and those living with a smoker(s) at home [8,9]. However, according to the results of the Korea youth risk behavior web-based survey (KYRBS), the exposure level to anti-tobacco media messages among Korean adolescents was consistently lower in the high-risk groups (smoking prevalence/exposure to anti-tobacco media messages in 2018: boys 9.1%/78.7%, senior grade students: 11.7%/78.3% in 12th grade) than in the low-risk groups (smoking prevalence/exposure to anti-tobacco media messages in 2018: girls 3.6%/86.3%, junior grade students: 0.7%/88.3% in 7th grade) since 2007 [10]. This suggests that strategies for delivering anti-tobacco media messages targeting the high-risk group of Korean adolescents may not have been fully considered so far. Therefore, it is necessary to reduce the gap in exposure to anti-tobacco media messages between the high- and low-risk groups as well as to find ways to deliver them more effectively to the high-risk groups.

In Korea, all schools must provide health education on the topic of smoking prevention in accordance with Article 9 (Health Care for Students) of the School Health Act. In Korea, schools should run safety education curriculums for seven areas (life safety, traffic safety, violence and personal safety, drug and Internet addiction, disaster safety, occupational safety, and first aid), and operate smoking prevention education as a field of drug and internet addiction [11]. Moreover, using the financial resources secured by the increase in tobacco prices from 2.5 USD (exchange rate: 1 USD = 1000 Korean Won) to 4.5 USD per cigarette pack in 2015, school-based smoking prevention education has been expanded to school-based smoking prevention programs [12,13]. According to the size of the school and the school stakeholders’ willingness to operate the program, the school-based smoking prevention program is divided into basic and advanced types, and the annual budget for each school is 500–2500 USD and 3000–12,000 USD, respectively [14]. It is essential for both basic and advanced types to operate smoking prevention education using standardized education programs. Additionally, managing smoking students and running school-based youth smoking cessation programs are essential in advanced type but optional in basic type [14]. Korea’s school-based smoking prevention program is conducted throughout the year, with various activities, such as campaigns, experience booths, and production and exhibition of works related to smoking prevention as well as education [15]. Students can be exposed to various educational materials, including anti-tobacco media messages, by participating in such programs. In particular, the main channels of exposure to anti-tobacco media messages for adolescents is TV or the Internet [10], and more than 80% of the schools participating in the school-based smoking prevention program showed videos on smoking prevention to the students in addition to the lectures [16]. Therefore, since all students in Korea participate in school-based smoking prevention education, using anti-tobacco media messages as educational content will not only increase the overall exposure level among students but also help to improve the exposure level in high-risk groups with low exposure levels. To test this hypothesis, it is necessary to accumulate evidence by evaluating the relationship between participation in school-based smoking prevention education and exposure to anti-tobacco media messages. Therefore, this study aimed to evaluate whether school-based smoking prevention education in Korea is related to exposure to anti-tobacco media messages, and whether education can contribute to reducing the gap in the exposure of anti-tobacco media messages between smoking vulnerable and non-vulnerable groups.

## 2. Materials and Methods

### 2.1. Study Sample

This study used the 2018 KYRBS conducted by the Korea Centers for Disease Control and Prevention. To produce nationally representative statistics concerning the health behaviors of Korean adolescents, stratified multistage probability sampling was used. Students from grades 7–12 anonymously completed a self-reported online survey. A total of 60,040 students from 800 sample schools (400 junior high and 400 high schools) participated (response rate = 95.6%) [10]. After excluding students who did not report information regarding their parental education, 59,410 students (30,045 boys and 29,365 girls) comprised the final sample. All study participants provided informed consent, and the KYRBS was approved by the Institutional Review Board of the Korea Centers for Disease Control and Prevention (2014-06EXP-02-P-A).

### 2.2. Measures

Individuals who had seen or heard anti-tobacco messages on television, radio, Internet, newspapers, billboards, posters, or magazines within the 12 months preceding the study were defined as people exposed to anti-tobacco messages on media within the last year. Participation in school-based smoking prevention education was evaluated through the answer (yes or no) to the following question: “During the past 12 months, have you ever participated in smoking prevention or cessation education at school (including formal education, broadcast education, large group education events, etc.)?”

Covariates included the following sociodemographic factors: gender, grade (7–12), stress level (high, moderate, or low), monthly alcohol drinking (yes or no), smoking status (never users, former users, current users (smoking <20 days/month), current users (smoking 20–29 days/month), or current users (smoking 30 days/month)), subjective academic performance (high, moderate, or low), perceived economic status (high, moderate, or low), parental education level, and residential area (metropolitan, small- or medium-sized city, or rural). Parental education was measured based on the highest level of education completed by the participants’ mother and father and was categorized into three groups: college or above for both parents, college or above for one of the parents, or high school or less for both parents.

### 2.3. Statistical Analysis

Weighted percentages of exposure level to anti-tobacco messages via mass media within the last one year were calculated. The following two methods were used to test the two research hypotheses (1. There is an association between exposure to anti-tobacco media messages and participation in school-based smoking prevention education, 2. There is a possibility of reducing the exposure gap between high- and low-risk groups for smoking when participating in education). First, logistic regression was used to evaluate the association between exposure to anti-tobacco media messages and school-based smoking prevention education. Second, we evaluated the possibility that participation in school-based smoking prevention education can contribute to reducing the gap in exposure to anti-tobacco media messages between high- and low-risk groups for smoking. To do so, we compared the differences in the exposure level within groups classified by sociodemographic characteristics depending on whether they participated in school-based smoking prevention education or not. High- and low-risk groups were classified based on smoking prevalence within each sociodemographic characteristic, and the difference between subgroups divided on the basis of participation in smoking prevention education was evaluated as the absolute difference between maximum and minimum values within the subgroups. In the questionnaire, there is no direct description that the contents of school-based smoking prevention education included the use of anti-tobacco media messages. Therefore, the results of the above two relevance between two factors are preliminary results and have limitations in their interpretation, and are useful to test the two research hypotheses. All analyses were performed using SPSS version 19.0 (IBM, Armonk, NY, USA), and a *p*-value < 0.05 was considered to indicate statistical significance. Complex SPSS sampling methods were used to accurately represent Korean adolescents.

## 3. Results

The characteristics of the study participants and their smoking prevalence are shown in Table 1. Of the total participants, 6.5% were current smokers. There was a significant difference in smoking prevalence in all variables except residential area, and the variables with particularly large differences in smoking prevalence within the group were gender, grade, subjective academic performance, and monthly alcohol drinking.

A total of 82.5% of the study participants were exposed to anti-tobacco media messages within the past year. The rate of exposure to media messages was higher among those who participated in school-based smoking prevention education within the past year (88.1%) than among those who did not (67.5%). The association between exposure to anti-tobacco media messages and experience of smoking prevention education within the past year was particularly prominent with an adjusted odds ratio of 3.41 (95% confidence interval [CI] 3.26–3.57). The logistic regression analysis revealed that boys, higher graders, current smokers (30 days/month), participants with low parental education level (both parents: high school or less), and inhabitants of rural areas demonstrated higher smoking prevalence and were less likely to be exposed to anti-tobacco media messages within the past year (Table 2).

After stratification according to participation in school-based smoking prevention education within the past year, the rates of exposure to anti-tobacco media messages were further investigated according to sociodemographic characteristics. In each subgroup, the lowest exposure rate to anti-tobacco media messages among individuals who participated in smoking prevention education within the past year (former smokers: 82.7%, 95% CI 90.1%–91.6%) was higher than the highest exposure rate among those who did not participate in any such education program (7th grade: 77.9%, 95% CI 75.9%–79.9%). The difference between the maximum and minimum exposure rate in each subgroup among adolescents who participated in smoking prevention education (8.2% p) was smaller than that among those without the experience of smoking prevention education (19.7% p). For most of the subgroups of sociodemographic characteristics, compared to those who did not participate in smoking prevention education, the exposure rate of those who participated was higher, and the exposure gap within subgroups was smaller (Table 3).

## 4. Discussion

The rate of exposure to anti-tobacco media messages within a year among Korean adolescents was 82.5%, and it was lower among current daily smokers and adolescents vulnerable to smoking (boys, higher grades, those whose parental education level was low, and residents of rural areas). This indicates that strategies for delivering anti-tobacco media messages targeting high-risk groups are not working, hindering the bridging of the gap in smoking prevalence between the high- and low-risk groups.

The Centers for Disease Control and Prevention (CDC) recommends mass media campaigns as best practices for comprehensive tobacco control programs. According to this guideline, mass media campaigns should reach at least 75% of the target audience each quarter of the year [17]. In the case of those who participated in school-based smoking prevention education, the minimum exposure rate for each subgroup was 82.7%, which met the CDC guideline and was higher than the maximum exposure rate (77.9%) for each subgroup that did not participate. School-based smoking prevention education can reduce the gap in exposure to anti-tobacco media messages between groups, especially in terms of subgroup characteristics such as gender, grade, and smoking status, which showed a high exposure gap. Although the gap between the subgroups based on differences in some social inequality-related factors (i.e., residential area) did not decrease according to participation in smoking prevention education, it was meaningful that the exposure level increased approximately 20% p in each subgroup.

The following reasons may explain why school-based smoking prevention education increases exposure to anti-tobacco media messages. First, participation in smoking prevention programs has been reported to affect smoking-related knowledge and attitude toward smoking [18,19], which can raise adolescents’ attention toward smoking prevention information. The idea that an individual’s attitude towards an object influences their attention to the object, leading them to selectively attend to information consistent with their attitude [20,21] supports that school-based smoking prevention education can influence students’ exposure to anti-tobacco media messages. Second, media messages are included in educational materials or reference materials of the school-based smoking prevention education curriculum. For example, the SENSE program, which is the Korean standard educational material for school-based smoking prevention education, includes several anti-tobacco media messages through videos, newspapers and posters [22]. In addition, the student health information center homepage operated by the Ministry of Education in Korea also provides various educational reference materials in the form of media messages [23]. In fact, more than 80% of the schools participating in the school-based smoking prevention program showed videos on smoking prevention to the students in addition to the lectures [16].

According to the logic of the model of CDC’s the National Tobacco Control Program, anti-tobacco media messaged contribute to reducing susceptibility to experimentation with tobacco products as one of the short-term outcomes of preventing smoking initiation [24]. Considering the two types of mechanisms mentioned above (increased interest, utilization of educational materials) that affect media exposure via school-based smoking prevention education, school-based smoking prevention education has the following advantages or expected outcomes in terms of exposure to anti-tobacco media messages. First, school-based smoking prevention education is a universal education conducted by all schools in Korea regardless of school level or geographical location. Given that most of this educational curriculum includes media content [16], school-based smoking prevention education can help to close the media exposure gap between the groups as well as increase exposure overall. These effects are expected to contribute to reducing the gap in smoking prevalence among high-risk groups for smoking based on social inequality factors (i.e., economic status, parental education levels) as well as based on demographic factors (i.e., gender, grade). Second, in school environments, the school nurse can reinforce media messages through brief counseling [7]. As school nurses are also school teachers in Korea, and teachers are trustworthy figures for students, they can impart additional knowledge about the messages that teachers use [7]. Third, students who have received smoking prevention education will be more interested in and will better remember the external mass media messages that are not included in the educational contents. These mechanisms will have a synergistic effect on the school-based smoking prevention program and the anti-tobacco media message effect. The National Cancer Institute report and Cochrane review also supported the effects of a combination of school-based programs and media messages. According to a report by the National Cancer Institute [2], mass media campaigns combined with school or community-based programs are effective in preventing smoking in adolescents. Cochrane database of systematic review also suggested that a combination of school-based components (e.g., school posters) and repetitive media messages appeared to contribute to successful campaigns [25]. Fourth, media message use in school-based smoking education programs can increase the level of media exposure in high-risk groups. In addition, smoking students have the advantage of participating in school-based smoking cessation programs or linking to community resources related to smoking cessation program such as local public health centers or quit line.

This study had several limitations. First, the reference period (last 12 months) used for the exposure experiences to anti-tobacco media messages was too long, potentially causing a recall bias. However, although there were differences in adolescent recall bias about exposure to anti-tobacco advertisements according to smoking experience or smoking amount [18,26], the number of adolescent smokers who may be affected by a recall bias was not large (6.5%). Therefore, recall bias is not expected to have a significant impact on the present results. Second, there is a limitation to the survey items because our study is based on secondary data from the KYRBS. For example, there was no information about the inclusion of media contents in school-based smoking prevention education. Therefore, the exact cause of the relationship between smoking prevention education and media message exposure cannot be explained. Third, this study was a cross-sectional study and could not confirm the causality because there was no temporal relationship between two factors. However, the effect size of the association between the two factors is large (OR = 3.41, 95% CI 3.26–3.57), and all subgroups participating in the school-based smoking prevention education consistently show the result of higher exposure to media messages. Considering that the main exposure channels of anti-tobacco media message in Korea are TV or the Internet, the TV or Internet penetration rate in Korea as of 2019 were 95.8% and 96.0% [27,28], which meant that the level of exposure to media messages would not be affected by other external factors such as residential area or individuals’ sociodemographic characteristics. Despite these limitations, this study is meaningful in that it suggests ways to increase exposure to anti-tobacco media messages and reduce the gap in exposure between smoking vulnerable and non-vulnerable groups by evaluating the relationship between school-based smoking prevention education and exposure to media messages. In particular, in the Korean environment where all schools operate school-based smoking prevention programs, the results of our study are meaningful, and the strategy of using media contents as educational material can be considered to maximize the effect. This strategy is easy to implement because all schools are participating in the school-based smoking prevention program, with approximately 80% of them already utilizing media contents [16]. Additionally, diversification of educational contents in school-based smoking prevention programs is helpful in forming students’ non-smoking intention [16]. Considering that the integration of sounds and images from multimedia with prior knowledge can increase the effectiveness of learning according to the cognitive theory of multimedia learning [29], the use of media contents in school-based smoking prevention education can be effective in preventing smoking among adolescents. In the case of the Real Cost Campaign in the United States, a strategy to increase message exposure by attaching posters on school bathrooms is proposed [30], but we have not yet found a study on the effect of reducing exposure gap among high- and low risk groups by using these strategies. Therefore, this study is a preliminary study that suggests the possibility of reducing the media messages exposure gap due to participation in school-based smoking prevention education or program. In the future, it is necessary to establish scientific evidence on causality evaluation between the two factors through longitudinal studies, and further research on specific application strategies is also needed.

## 5. Conclusions

Approximately eighty percent of Korean adolescents were exposed to anti-tobacco media messages within the one year surveyed in this study. However, notably, students who were currently smoking, and those who did not smoke but were susceptible to smoking were less likely to be exposed to anti-tobacco messages. Participation in school-based smoking prevention education showed a significant relationship with exposure to media messages and reduced the gap in media messages exposure rate among high- and low-risk groups for smoking. This relationship can be used as a strategy to improve the overall rate of exposure to media messages and reduce the differences in exposure rate between groups based on sociodemographic traits.

## Figures and Tables

**Table 1:** Characteristics of participants and current smoking prevalence according to characteristics.

Category	Full Sample		Current Smoker (Weighted %, 95% CI)
	N	Weighted % ^a^	
Total	59,410	100.0	6.5 (6.1–6.9)
Main factors			
Exposure to anti-tobacco media messages (within a year)			
Yes	49,103	82.5	6.1 (5.7–6.5)
No	10,307	17.5	8.2 (7.5–8.9)
Participation in smoking prevention education (within a year)			
Yes	43,934	72.6	6.2 (5.8–6.6)
No	15,476	27.4	7.3 (6.6–8.0)
Sociodemographic factors			
Gender			
Boys	30,045	51.9	9.1 (8.6–9.7)
Girls	29,365	48.1	3.6 (3.3–3.9)
Grade			
7th	9789	14.6	0.7 (0.5–1.0)
8th	10,011	15.7	3.1 (2.7–3.6)
9th	10,159	16.2	4.6 (4.0–5.1)
10th	9151	15.9	7.5 (6.7–8.3)
11th	9926	17.8	9.2 (8.4–10.0)
12th	10,374	19.8	11.7 (10.6–12.7)
Stress level			
High	24,039	40.4	7.6 (7.0–8.1)
Moderate	24,434	41.4	5.7 (5.3–6.2)
Low	10,937	18.2	5.8 (5.3–6.4)
Monthly alcohol drinking			
Yes	9467	16.7	27.4 (26–28.7)
No	49,943	83.3	2.3 (2.1–2.5)
Subjective academic performance			
High	23,182	38.7	3.9 (3.6–4.3)
Moderate	17,417	29.5	5.2 (4.8–5.7)
Low	18,811	31.8	10.7 (10.1–11.4)
Perceived economic status			
High	23,981	40.8	6.0 (5.5–6.5)
Middle	27,638	46.2	6.0 (5.6–6.4)
Low	7791	12.9	9.9 (9.0–10.8)
Parental education levels			
College or above (both parents)	23,981	41.8	4.9 (4.4–5.3)
College or above (one of parent)	12,855	21.7	7.1 (6.6–7.7)
High school or less (both parents)	22,574	36.5	7.9 (7.3–8.6)
Area			
Urban (Metropolitan city)	26,395	42.8	6.1 (5.5–6.7)
Urban (small- &medium-sized city)	28,565	51.1	6.7 (6.1–7.3)
Rural	4450	6.1	7.2 (5.2–9.2)

Abbreviations: CI, confidence interval. ^a^ Rows may not add up to 100% due to rounding off.

**Table 2:** Evaluation of exposure to anti-tobacco media messages within a year.

Category	Exposure to Anti-Tobacco Media Messages within a Year		
	Weighted % (95% CI)	Unadjusted OR (95% CI)	Adjusted OR ^a^ (95% CI)
Main factors			
Participation in smoking prevention education (within a year)			
Yes	88.1 (87.8–88.5)	**3.58 (3.42–3.75)**	**3.41 (3.26–3.57)**
No	67.5 (66.5–68.4)	Ref	Ref
Sociodemographic factors			
Gender			
Boys	78.9 (78.2–79.5)	**0.59 (0.56–0.62)**	**0.61 (0.58–0.64)**
Girls	86.4 (85.9–86.9)	Ref	Ref
Grade			
7th	88.3 (87.6–89.1)	Ref	Ref
8th	84.1 (83.3–84.9)	**0.70 (0.64–0.76)**	**0.71 (0.65–0.78)**
9th	82.8 (81.9–83.7)	**0.64 (0.58–0.70)**	**0.68 (0.62–0.75)**
10th	82.0 (81.1–83.0)	**0.60 (0.55–0.66)**	**0.71 (0.65–0.78)**
11th	80.9 (79.9–81.8)	**0.56 (0.51–0.61)**	**0.67 (0.61–0.73)**
12th	78.4 (77.4–79.5)	**0.48 (0.44–0.53)**	**0.65 (0.59–0.71)**
Stress level			
High	82.1 (81.5–82.7)	**1.08 (1.02–1.15)**	1.01 (0.95–1.07)
Moderate	83.5 (82.9–84.2)	**1.20 (1.13–1.27)**	**1.16 (1.09–1.23)**
Low	80.9 (80.1–81.7)	Ref	Ref
Monthly alcohol drinking			
Yes	79.8 (78.8–80.8)	**0.81 (0.76–0.86)**	1.01 (0.94–1.09)
No	83.0 (82.6–83.5)	Ref	Ref
Smoking status			
Never	83.6 (83.1–84.0)	Ref	Ref
Former	75.0 (73.7–76.3)	**0.59 (0.55–0.64)**	**0.74 (0.68–0.79)**
Current (<20 days/month)	80.0 (77.9–82.0)	**0.79 (0.69–0.89)**	0.95 (0.82–1.09)
Current (20–29 days/month)	80.1 (75.9–84.3)	0.79 (0.61–1.03)	1.03 (0.77–1.38)
Current (30 days/month)	75.5 (73.1–77.8)	**0.61 (0.53–0.69)**	**0.85 (0.74–0.98)**
Subjective academic performance			
High	84.6 (84.0–85.2)	Ref	Ref
Moderate	83.4 (82.8–84.0)	**0.92 (0.87–0.97)**	0.97 (0.92–1.03)
Low	79.1 (78.4–79.8)	**0.69 (0.65–0.73)**	**0.80 (0.75–0.85)**
Perceived economic status			
High	82.7 (82.1–83.3)	Ref	Ref
Middle	82.7 (82.1–83.2)	1.0 (0.95–1.05)	1.04 (0.99–1.10)
Low	81.1 (80.0–82.1)	**0.90 (0.83–0.96)**	1.02 (0.95–1.10)
Parental education levels			
College or above (both parents)	83.2 (82.6–83.8)	ref	ref
College or above (one of parent)	83.7 (83.0–84.4)	1.04 (0.98–1.11)	1.09 (1.03–1.16)
High school or less (both parents)	81.0 (80.4–81.6)	**0.86 (0.82–0.91)**	**0.91 (0.86–0.96)**
Area			
Urban (metropolitan city)	82.9 (82.2–83.6)	Ref	Ref
Urban (small- and medium-sized city)	82.2 (81.6–82.9)	0.96 (0.89–1.02)	0.98 (0.92–1.03)
Rural	81.6 (79.9–83.2)	0.92 (0.81–1.03)	**0.88 (0.78–0.99)**

Abbreviations: CI, confidence interval; OR, odds ratio. ^a^ Adjusted for sex, grade, stress level, subjective academic performance, monthly alcohol drinking, smoking status, smoking prevention program within a year, area, perceived economic status, and parental education levels. Bold characters indicate significant associations (*p* < 0.05).

**Table 3:** Exposure to anti-tobacco media messages according to the participation of school-based smoking prevention education within a year.

Category	Participation of Smoking Prevention Education (within a Year)			
	Yes		No	
	Weighted % (95% CI)	Difference ^a^ (% *p*)	Weighted % (95% CI)	Difference ^a^ (% *p*)
Summary of results from all subgroups		8.2		19.7
Maximum ^b^	90.9 (90.1–91.6)		77.9 (75.9–79.9)	
Minimum ^b^	82.7 (81.4–83.9)		58.2 (55.6–60.8)	
Sociodemographic factors				
Gender		5.3		11.0
Boys	85.5 (85.0–86.0)		62.5 (61.3–63.8)	
Girls	90.8 (90.4–91.2)		73.5 (72.4–74.6)	
Grade		3.6		13.1
7th	90.9 (90.1–91.6)		77.9 (75.9–79.9)	
8th	87.7 (86.9–88.5)		69.9 (67.9–71.9)	
9th	87.4 (86.6–88.2)		67.0 (64.6–69.5)	
10th	88.2 (87.3–89.0)		67.0 (65.1–68.9)	
11th	87.4 (86.7–88.2)		65.1 (63.3–67.0)	
12th	87.3 (86.4–88.2)		64.8 (63.0–66.5)	
Stress level		1.5		5.3
High	88.0 (87.5–88.5)		67.0 (65.7–68.3)	
Moderate	88.7 (88.2–89.2)		69.5 (68.2–70.8)	
Low	87.2 (86.4–87.9)		64.2 (62.3–66.1)	
Monthly alcohol drinking		2.2		2.6
Yes	86.3 (85.4–87.2)		65.4 (63.4–67.4)	
No	88.5 (88.1–88.9)		68.0 (67.0–69.0)	
Smoking status		6.2		10.9
Never	88.9 (88.6–89.3)		68.9 (67.9–69.8)	
Former	82.7 (81.4–83.9)		58.2 (55.6–60.8)	
Current (<20 days/month)	84.2 (82.1–86.3)		69.1 (64.3–73.8)	
Current (20–29 days/month)	86.0 (81.6–90.3)		65.9 (56.5–75.3)	
Current (30 days/month)	83.6 (81.4–85.9)		59.3 (54.7–64.0)	
Subjective academic performance		4.4		4.3
High	89.8 (89.3–90.3)		67.7 (66.3–69.2)	
Moderate	88.7 (88.1–89.3)		69.7 (68.4–7.01)	
Low	85.4 (84.7–86.0)		65.4 (64.1–66.8)	
Perceived economic status		1.8		2.0
High	88.6 (88.1–89.1)		67.1 (65.8–68.5)	
Middle	88.1 (87.6–88.6)		68.2 (67.0–69.4)	
Low	86.8 (85.9–87.7)		66.2 (64.0–68.4)	
Parental education levels		2.5		2.9
College or above (both parents)	89.0 (88.4–89.5)		67.6 (66.3–69.0)	
College or above (one parent)	89.1 (88.4–89.7)		69.2 (67.6–70.8)	
High school or less (both parents)	86.6 (86.1–87.2)		66.3 (64.9–67.7)	
Area		2.6		1.5
Urban (metropolitan city)	88.8 (88.3–89.3)		67.0 (65.4–68.5)	
Urban (small- and medium-sized city)	87.8 (87.3–88.4)		68.0 (66.7–69.2)	
Rural	86.2 (84.8–87.6)		66.5 (62.6–70.3)	

^a^ Difference means the value of maximum–minimum in the same category. ^b^ The maximum or minimum values among all subgroups were the 7th-grade group or former smokers, respectively.
